# Predicting mortality of residents at admission to nursing home: A longitudinal cohort study

**DOI:** 10.1186/1472-6963-11-86

**Published:** 2011-04-20

**Authors:** Ingibjörg Hjaltadóttir, Ingalill Rahm Hallberg, Anna Kristensson Ekwall, Per Nyberg

**Affiliations:** 1 Department of Health Sciences, Lund University, Lund, Sweden; 2 Faculty of Nursing, University of Iceland, Reykjavik, Iceland; 3 Internal medical Services, The National University Hospital of Iceland, Reykjavik, Iceland; 4 The Swedish Institute for Health Sciences, Lund University, Lund, Sweden

## Abstract

**Background:**

An increasing numbers of deaths occur in nursing homes. Knowledge of the course of development over the years in death rates and predictors of mortality is important for officials responsible for organizing care to be able to ensure that staff is knowledgeable in the areas of care needed. The aim of this study was to investigate the time from residents' admission to Icelandic nursing homes to death and the predictive power of demographic variables, health status (health stability, pain, depression and cognitive performance) and functional profile (ADL and social engagement) for 3-year mortality in yearly cohorts from 1996-2006.

**Methods:**

The samples consisted of residents (N = 2206) admitted to nursing homes in Iceland in 1996-2006, who were assessed once at baseline with a Minimum Data Set (MDS) within 90 days of their admittance to the nursing home. The follow-up time for survival of each cohort was 36 months from admission. Based on Kaplan-Meier analysis (log rank test) and non-parametric correlation analyses (Spearman's rho), variables associated with survival time with a p-value < 0.05 were entered into a multivariate Cox regression model.

**Results:**

The median survival time was 31 months, and no significant difference was detected in the mortality rate between cohorts. Age, gender (HR 1.52), place admitted from (HR 1.27), ADL functioning (HR 1.33-1.80), health stability (HR 1.61-16.12) and ability to engage in social activities (HR 1.51-1.65) were significant predictors of mortality. A total of 28.8% of residents died within a year, 43.4% within two years and 53.1% of the residents died within 3 years.

**Conclusion:**

It is noteworthy that despite financial constraints, the mortality rate did not change over the study period. Health stability was a strong predictor of mortality, in addition to ADL performance. Considering these variables is thus valuable when deciding on the type of service an elderly person needs. The mortality rate showed that more than 50% died within 3 years, and almost a third of the residents may have needed palliative care within a year of admission. Considering the short survival time from admission, it seems relevant that staff is trained in providing palliative care as much as restorative care.

## Background

Knowledge about predictors of mortality of nursing home residents is sparse, in particular regarding whether the survival time has been shortening in recent decades due to more restrictive admission criteria. Additionally, knowledge about predictors of mortality is needed to provide appropriate care and ensure that the staff are knowledgeable in the areas of care that are most needed. Officials organizing care and services for older people also need to be aware of shifts in the need for services that may take place over time due to changes in, for instance, financial resources.

Several factors have been found to predict mortality at admission to a nursing home. Three studies investigating admission status have all reported cancer or history of malignancy to be a predictor of mortality (UK; N = 308)[1] (US; n = 100,669)[2] (UK; N = 1557) [3]. Predictors reported by two studies have been related to physical disability [1,2], problems with eating [2,3] and use of medication [1,3]. Other predictors reported have been infection at admittance [1], pressure ulcer, bowel incontinence [2], age, male gender, sleep disturbance, where admitted from, and respiratory disease [3]. The mean survival time for newly admitted nursing home residents differs and has been reported as 76 days for men and 134 days for women [1] or 5.9 years for both genders [3].

Study design, time of assessment and the delay in assessment from time of admittance are most likely factors that affect the outcome. For instance, predictors of mortality at admission in relation to predictors of mortality among residents living in a nursing home for more than one year seemingly differ [2]. There is no consensus on how to assess for predictors of mortality and therefore comparison may be difficult. Thus, there is not yet a coherent body of knowledge about factors predicting mortality at admission with sufficiently clear implications for planning and nursing care. The current knowledge base has weaknesses both in terms of the low number of studies, the methods used to identify predictors, and whether there have been changes in mortality over time.

Death may not be openly discussed in nursing homes, even though an increasing number of deaths occur there [4]. However, a short survival time underlines the need for knowledge of palliative care in nursing homes [5]. It has been pointed out that the framework of palliative care may be appropriate not only for older people at the very end of life but also for those receiving long-term care [6]. Research has shown a lack of symptom treatment and access to palliative care for dying residents, resulting in their suffering [7,8]. Furthermore, researchers have pointed out several internal factors that are challenging when delivering palliative care in nursing homes. These include the staffs' knowledge on how to provide palliative care, their attitude toward palliative care, staffing levels, lack of physician support, lack of privacy, family expectations for care, and the hospitalization of residents [9].

The official policy in Iceland is to enable older people to stay at home as long as possible [10]. The long term care available is home care, including both domestic help and nursing care. Those needing around-the-clock care either go first into residential care or directly to a nursing home, depending on their needs. A percentage of the resident's pension goes toward their upkeep; otherwise the service is government funded [10]. A short standardized preadmission assessment is used to prioritize who is to be admitted into a residential or nursing home [11].

In Iceland there are 62 nursing homes, with room for about 2500 residents or about 8% of those in Iceland aged 67 and older (retirement age in Iceland) [10,12]. A nursing home in Iceland is an institution or ward where nursing care is provided to the residents 24 hours a day. The care is delivered by registered nurses, licensed practical nurses and nursing assistants. On average 4.1-5.0 nursing hours are provided per patient per 24-hour period, and the nurse-patient ratio is 0.88. Registered nurses constitute 18% of the staff, licensed practical nurses 20%, other professionals 1%, and nursing assistants 61% [10]. Assistance with the activities of daily living (ADL), moving about and recreation is provided at the nursing home. A physician visits the nursing home 3-5 times a week as well as being on call around the clock for emergencies. Physiotherapy is provided at most nursing homes, and some also provide occupational therapy. End of life care is provided in the nursing homes and most of the residents die there; as few as 20% of residents move to a hospital before death [13]. A few nursing homes provide respite care or rehabilitation and nursing homes also provide care for people younger than 67 years old [10]. An earlier analysis of the sample used in this study showed that 52.7% to 67.1% of the cohorts admitted to Icelandic nursing homes in the period 1996-2006 were women, and the mean age was from 80.1 to 82.8 years. Those with pain every day ranged from 29.6% to 40.9%, and 16.2% to 31% had signs of depression. Bladder incontinence ranged from 17.8% to 41.6% and bowel incontinence from 6.5% to 20%. Residents having short-term memory problems varied from 49.2% to 75.7%, and those needing extensive assistance or who were totally dependent on help in getting to the toilet ranged from 20.3% to 54.8% [14].

Knowledge of factors influencing mortality, the average length of survival and residents' health status at admission are critical to managers and health officials involved in nursing home care. The staff's knowledge has also been shown to affect resident's quality of care [15]. The main goal of the nursing care of residents may not be to prolong their life [16] but, rather, to add quality to their lives. The present study will contribute knowledge about changes and the trend over time in residents' health conditions and factors associated with the mortality of those moving to nursing homes.

The aim of this study was to investigate the time from residents' admission to Icelandic nursing homes to death and the predictive power of demographic variables, health status (health stability, pain, depression and cognitive performance) and functional profile (ADL and social engagement) for 3-year mortality in yearly cohorts from 1996-2006.

## Methods

### Sample

The sample consisted of newly admitted nursing home residents in Iceland for each year for the period 1996-2006 who had been assessed with the Minimum Data Set (MDS) within 90 days of their admittance to the nursing home (n = 2206) to capture their state of health at admission. Residents assessed more than 90 days after admittance were not included in the sample (n = 2527). During these 11 years a total of 4733 residents were assessed; however, according to official data 4700 were admitted to nursing homes, leaving 33 extra assessments possibly due to residents moving between nursing homes [17]. The study sample represented 46.6% of the total admissions over the years. The admission criteria were not changed over the research period. The data were accessed from a central database stored by the Icelandic Ministry of Health. The database also stores the residents' time of death retrieved from the national registry where all deaths are registered. The follow-up time for time of death for each cohort was 3 years from admission.

### Instrument and procedure

The Minimum Data Set (MDS) is a part of the Resident Assessment Instrument (RAI) and is used to assess functioning and health care needs of nursing home residents [18,19]. Since 1996 the MDS assessment of all nursing home residents has been mandatory in accordance with a regulation set by the Icelandic Minister of Health [10]. The MDS assessment has been used internationally for research purposes but was originally designed as a clinical tool intended to improve care [18,19]. The Minimum Data Set for nursing homes (MDS), version 2.0, has 21 sections with about 350 clinical data elements. The MDS instrument is considered to be an extensive, reliable and valid instrument [20-22] and has enabled comparison between countries and institutions. The assessment is carried out by registered nurses, with physiotherapists and doctors participating, and is based on observation, clinical documentation and interviews with the residents and or their family members. Researchers have reported adequate inter-rater reliability (Kappa > 0.6) for 85% of the MDS data elements [23]. Ten of the variables used in this study have been reported to have moderate to perfect agreement [24]. The variables from the MDS assessment used in this analysis were demographic variables (age, gender, year of admittance, place admitted from, and month of death) and scores from scales and indices developed especially for the MDS which can be used to monitor changes over time.

The *CHESS Scale *(Changes in Health, End-stage disease and Signs and Symptoms) ranges from 0 meaning that the individual is stable to a score of 5 indicating unstable health, risk of mortality, hospitalization, pain, caregiver stress and poor self-rated health. The scale is known to be a strong predictor of mortality [25].

The *Pain Scale *(PS) ranges from 0 indicating no pain to a score of 3 meaning that the resident is in severe (horrible/excruciating) pain [26]. It has been reported valid in detecting pain in nursing home residents [26].

The *Depression Rating Scale *(DRS) is a 15-point scale ranging from 0-14. A score of 0 means no indication of depression. A score of 3 indicates mild depression and a score of 14 very severe depression [27]. Researchers have reported excellent sensitivity and acceptable specificity; however, there is a need for further testing [27].

The *Cognitive Performance Scale *(CPS) ranges from 0 indicating that the resident is cognitively intact to 6 indicating severe cognitive impairment. The scale correlates moderately well with the Mini-Mental State Examination [28].

The *ADL long scale *is a 29-point scale, with a higher score indicating a greater need for assistance in the ADL activities (scale range 0-28) [20]. The scale has been reported to be sensitive to change [29].

The *Index of Social Engagement *(ISE) ranges from 0 meaning severe withdrawal from social engagement to 6 indicating that the resident has much initiative and participates in social activities. The range 0-2 has been described as indicating low social engagement compared to those with scores 3-6 [30].

### Statistical methods

This study follows cohorts of residents admitted each year from 1996-2006. Descriptive and analytical statistics were used. The Mann-Whitney U-test with a Bonferroni correction for multiple comparisons was used for ordinal data and skewed continuous data. The chi-square test and the chi-square test for trend were used for nominal data. Survival analysis, comprising 36 months from admission, was performed, controlling for age. The association between survival and categorical potential risk variables (gender, age-group, where admitted from, year of admission) were analysed using Kaplan-Meier analysis (log-rank test). The association between survival and ordinal risk variables (RAI scales) was analysed by non-parametric correlation analyses (Spearman's rho). Variables in these analyses associated with survival time with a p-value < 0.05 were entered into a multivariate Cox regression model (Backward stepwise; Likelihood-ratio) [31]. The Cox regression was performed controlling for age and controlling for age and gender. No multi-collinearity problem was detected. Partial correlation was used to further explore the relationship between social engagement and survival time while controlling for ADL functioning and health stability. The ADL Long scale was collapsed into 4 groups (scores 0-3, 4-9, 10-17, 18-28) in order to have fewer groups in the Cox regression. A limitation in the analysis is due to that nursing home as a variable was not possible to obtain and thus interpretation of the results should be made with that in mind. Data analysis was conducted with the software program SPSS version 17 and PASW Statistics 18.

### Ethical approval

This research project was approved by the Icelandic National Bioethics Committee (07-0330-S1) and the Data Protection Authority of the Icelandic Ministry of Justice (2007020171).

## Results

Of the total sample (N = 2206) 59.8% were women and the mean age was 82.5 years (SD 7.60). Women were older than men at admission (p < 0.0001) (n = 1319). Their mean age was 82.9 years (SD 7.80), and the mean age of men (n = 887) was 81.4 years (SD 8.20). Variation in sample size is shown in table 1. Residents were admitted from home (n = 1019, 46.8%), hospital (n = 805, 37.0%) other residential or nursing homes (n = 201, 9.2%) and assisted-living facilities (n = 151, 6.9%). A significant difference was not found within cohorts in gender and the average age of the excluded residents (assessed later than 90 days from admission), compared with the sample. The excluded residents' mean age ranged in each cohort from 80.6 to 82.8 years (NS), and the proportion of women was from 60.5% to 67.7% (NS) [14].

**Table 1:** Number of residents in each cohort (1996-2006) dying within the 1st, 2nd and 3rd years from admittance, 3-year mortality and median survival in months

Sample N = 2206		Died within 1st year n = 636 (28.8%)	Died within **2nd year**^**#**^ n = 322 (14.6%)	Died within ** 3rd year**^**##**^ n = 213 (9.7%)	3-year mortality*	Median survival (Q_1,_Q_3_)
**Year**	Cohorts n (%)**	**n (%)**	**n (%)**	**n (%)**	**%**	**Months**

1996	58 (19.9)	19 (32.8)	8 (13.8)	11 (19.0)	65.5	26.5 (10.0, 44.3)
1997	73 (22.1)	18 (24.7)	11 (15.1)	9 (12.3)	52.1	34.0 (14.0, 62.0)
1998	42 (13.1)	11 (26.2)	6 (14.3)	8 (19.0)	59.5	29.0 (9.0, 54.5)
1999	197 (54.3)	56 (28.4)	18 (9.1)	23 (11.7)	49.2	36.0 (10.5, 55.5)
2000	146 (40)	42 (28.8)	23 (15.8)	19 (13.0)	57.5	28.5 (11.0, 48.0)
2001	142 (39.2)	42 (29.6)	24 (16.9)	17 (12.0)	58.5	27.5 (8.0, 36.0)
2002	149 (28.9)	40 (26.8)	20 (13.4)	22 (14.8)	55.0	31.0 (9.5, 38.0)
2003	266 (52.9)	70 (26.3)	50 (18.8)	34 (12.8)	68.1	30.5 (10.8, 36.0)
2004	434 (69.7)	116 (26.7)	69 (15.9)	70 (16.1)	NA	NA
2005	401 (84.1)	106 (26.4)	93 (23.2)	NA	NA	NA
2006	298 (54.4)	116 (38.9)	NA	NA	NA	NA
Total	2206 (46.6)	636 (28.8)	NA	NA	NA	NA

*Chi-square test for trend showed no significant difference in mortality between cohorts

** Number of residents in each cohort and % of the total number of residents assessed that year [14]

# Years 1996-2005; ## Years 1996-2004; NA = Not Applicable

The median survival time for those admitted from 1996 to 2003 was 31 months (IQR 40), and 53.1% (n = 1171) of the residents died during the first 3 years of living in a nursing home. Residents dying in the first year were 28.8% (n = 636) of the total; 14.6% (n = 322) died during the second year, and 9.7% (n = 213) died during the third year. In the different cohorts residents dying in the first year ranged from 24.7% to 38.9% of the total, in the second year 9.1% to 23.2% and in the third year 11.7% to 19.0%. Residents living longer than 3 years were 46.9% (n = 1035) of the total. No significant difference was seen in median survival and mortality rates between cohorts (Table 1).

The median score of the sample for health stability was 1 (IQR 2), for pain 1 (IQR 2), for depression 1 (IQR 2), for cognitive performance 2 (IQR 2), for ADL performance 9 (IQR 14) and for social engagement 2 (IQR 4). The health of residents dying in the first year after admission to a nursing home was more unstable (p < 0.001) and their ADL performance was worse (p < 0.001) at admittance than for those dying in the second and third year. They also had more pain (p = 0.02) than those dying in the second year and were more depressed (p = 0.009) and less involved in social engagement (p < 0.001) than those dying in the third year. The health of residents dying in the second year after admission was less stable than for those dying in the third year (p < 0.001). Residents living more than 3 years from admission had better ADL performance (P = 0.004), better cognitive performance and were more involved in social engagement (p < 0.001) than those dying in the first to third year from admittance. Their health was more stable than of those dying in the first and second year (p < 0.001), and they were less depressed and in less pain than those dying in the first year (p < 0.001). The median values, Q_1 _and Q_3 _of the variables for those dying in the first to third year or lived longer than 3 years are shown in Table 2.

**Table 2:** Median values, Q_1 _and Q_3 _at admission for the CHESS Scale, Pain Scale, Depression Rating Scale, Cognitive Performance Scale, ADL Long Scale and the Index of Social Engagement for residents dying within the 1st, 2nd and 3rd years or living longer than 3 years from admission to a nursing home

Scale	Died within the 1st year n = 636 (28.8%)	Died within the 2nd year n = 322 (14.6%)	Died within the 3rd year n = 213 (9.7%)	Lived longer than 3 years n = 1035 (46.9%)
	**Median (Q_1,_Q_3_)**	**Median (Q_1,_Q_3_)**	**Median (Q_1,_Q_3_)**	**Median (Q_1,_Q_3_)**

CHESS Scale (range 0-5)	2 (1, 3)	1 (0, 2)	1 (0, 2)	1 (0, 2)
Pain Scale (range 0-5)	1 (0, 2)	1 (0, 2)	1 (0, 2)	1 (0, 2)
Depression Rating Scale (range 0-14)	1 (0, 3)	1 (0, 2)	1 (0, 2)	1 (0, 2)
Cognitive Performance Scale (range 0-6)	3 (1, 5)	3 (1, 4)	3 (1, 3)	2 (1, 3)
ADL long scale (range 0-28)	14 (7, 23)	10 (5, 15)	9 (3, 14)	6 (2, 13)
Index of Social Engagement (range 0-6)	1 (0, 3)	2 (0, 4)	2 (0, 4)	3 (1, 4)
Age	84 (80, 89)	83 (78, 88)	84 (79, 88)	82 (77, 86)

The number of males and females who died within the first three years (%) and of those who survived longer than 3 years are shown by scale values for the CHESS Scale, Pain Scale, Depression Rating Scale, Cognitive Performance Scale, ADL Long Scale and the Index of Social Engagement in table 3. The death rate for males and females increased with higher scores for the CHESS Scale, Depression Rating Scale, Cognitive Performance Scale, and the ADL Long Scale. In contrast, the death rate decreased with higher scores for the Index of Social Engagement, i.e. increased activity (table 3).

**Table 3:** Number of males and females who died within first three years (%) and survived longer than 3 years by scale values for the CHESS Scale, Pain Scale, Depression Rating Scale, Cognitive Performance Scale, ADL Long Scale and the Index of Social Engagement

Scale	Gender	Died during years 1-3 n (%)	Lived longer than 3 years	Total n	Scale	Gender	Died during years 1-3 n (%)	Lived longer than 3 years	Total n
**CHESS Scale**					**Cognitive Performance Scale**				
	
0-1	Male	274 (51.4)	259	533	0-1	Male	154 (48.4)	164	318
	Female	325 (39.3)	502	827		Female	216 (41.3)	307	523
2-3	Male	203 (72.0)	79	282	2-3	Male	227 (65.2)	121	348
	Female	207 (54.5)	173	380		Female	233 (46.3)	270	503
4-5	Male	64 (91.4)	6	70	4-6	Male	160 (73.1)	59	219
	Female	94 (87.0)	14	108		Female	177 (61.2)	112	289
**Pain Scale**					**Index of Social Engagement**				
	
0	Male	205 (58.9)	143	348	0-2	Male	383 (69.8)	166	549
	Female	150 (41.1)	215	365		Female	379 (55.6)	303	682
1	Male	161 (57.9)	117	278	3-4	Male	108 (50.2)	107	215
	Female	192 (47.1)	216	408		Female	160 (41.1)	229	389
2	Male	175 (67.6)	84	259	5-6	Male	50 (41.3)	71	121
	Female	284 (52.4)	258	542					
**Depression Rating Scale**					**ADL long scale**				
	
0-2	Male	412 (59.1)	285	697	0-3	Male	99 (45.8)	117	216
	Female	450 (45.7)	535	985		Female	115 (32.4)	240	355
3-8	Male	113 (66.1)	58	171	4-9	Male	119 (58.3)	85	204
	Female	153 (53.1)	135	288		Female	152 (41.4)	215	367
9-14	Male	14 (93.3)	1	15	10-17	Male	153 (66.2)	78	231
	Female	21 (55.3)	17	38		Female	168 (54.0)	143	311
					18-28	Male	170 (72.6)	64	234
						Female	191 (67.7)	91	282

**Figure 1:**
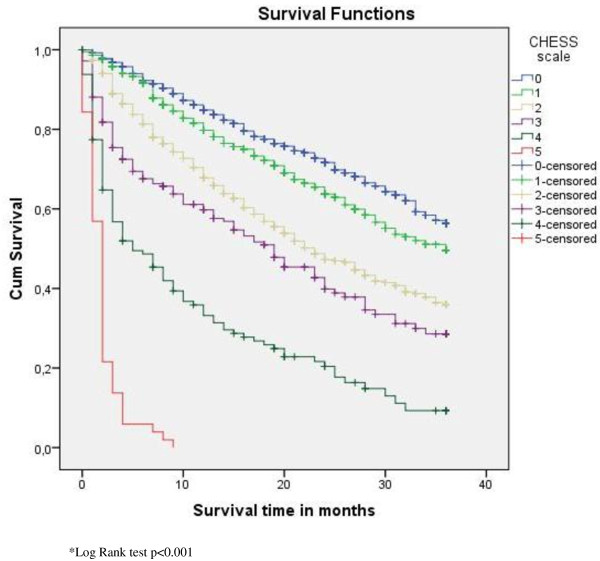
**Survival curves for the CHESS scale**.

**Figure 2:**
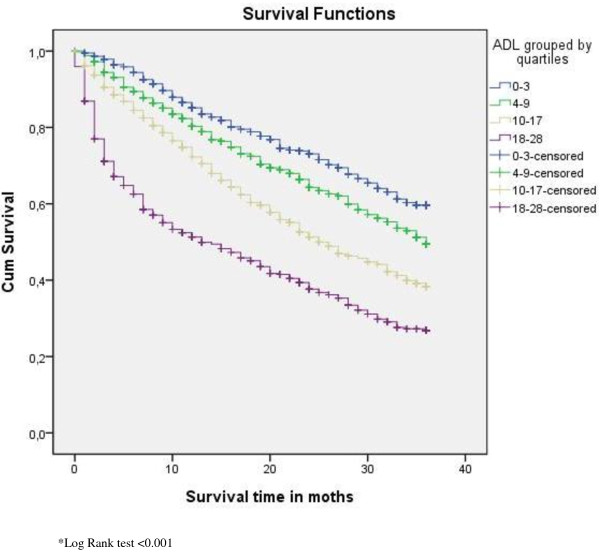
**Survival curves for residents' ADL scores by quartiles**.

**Figure 3:**
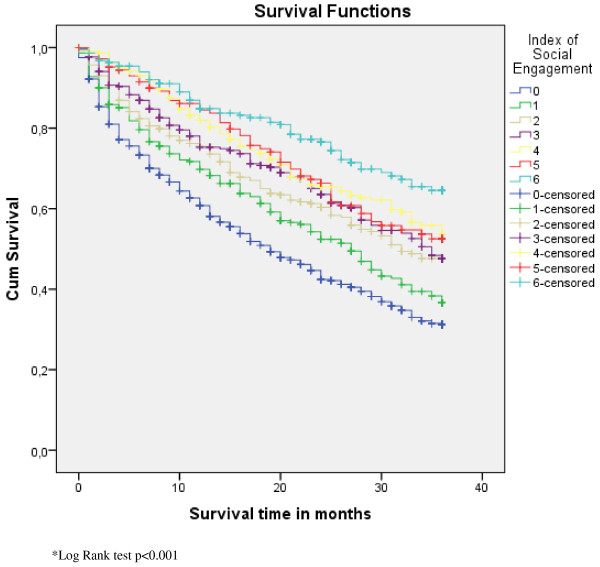
**Survival curves for the Index of Social Engagement scale**.

Figures 1, 2 and 3 display the survival curves for the CHESS scale, the ADL scale and the index of social engagement (log rank test p < 0.001).

The probability of dying increased with age, male gender, admitted from a hospital, more disability in ADL function and less stability in health. Predictors of mortality are presented in table 4 adjusted for age and in table 5 adjusted for age and gender. The probability of dying decreased with a higher ability to participate in social engagement. There was also a weak but significant partial correlation between social engagement and survival time whilst controlling for ADL functioning and health stability (r = 0.062, n = 2204, p < 0.004), with more social engagement being associated with longer survival. The zero order correlation (r = 0.191) suggested that controlling for ADL capacity and health stability had some effect on the relationship of social engagement and survival time. The ADL performance score from 4-9 was not a significant predictor of mortality, whereas higher scores were. The changes in health score were significant in all categories except the lowest score of 1. A higher score (2-5), i.e. more instability in health, meant a higher hazard ratio. A score of 5 meant a 15.7 times greater likelihood of dying than the reference group, i.e. than those with a score of 0. The scores 0-2 (withdrawal) on social engagement were significant predictors of mortality (Tables 4-5).

**Table 4:** Predictors of mortality (controlled for age*) **

		95% Confidence Interval for Exp(B)		
	**Hazard ratio - Exp(B)**	**Lower**	**Upper**	**p-value**

Gender male	1.52	1.34	1.73	< 0.001
ADL Long Scale^#^				< 0.001
1 = 0-3	1			
2 = 4-9	1.17	0.95	1.43	0.140
3 = 10-17	1.33	1.08	1.63	0.006
4 = 18-28	1.80	1.46	2.23	< 0.001
The Changes in Health Scale^##^				< 0.001
0	1			
1	1.18	0.98	1.41	0.078
2	1.61	1.35	1.93	< 0.001
3	2.17	1.71	2.75	< 0.001
4	3.89	3.03	4.99	< 0.001
5	16.12	11.42	22.75	< 0.001
Index of Social Engagement^###^				0.006
6	1			
5	1.37	0.95	1.98	0.094
4	1.20	0.87	1.66	0.273
3	1.33	0.97	1.83	0.077
2	1.51	1.11	2.07	0.010
1	1.63	1.19	2.22	0.002
0	1.65	1.23	2.21	0.001
Admitted from				0.011
Private home, with and without home help	1			
Board and care/assisted living/group home	1.09	0.84	1.41	0.512
Nursing home/nursing ward	1.11	0.89	1.38	0.361
Acute care hospital/ rehabilitation hospital	1.27	1.10	1.47	0.001

*Cox regression was performed controlling for age in four age groups (50-79, 80-84, 85-89, 90-104)

**Variables entered into the Cox regression were: gender, ADL Long Scale, CHESS, ISE, admitted from, pain scale, CPS and DRS.

# Score 0 = independent or only needs supervision; Score 28 = severe impairment in all four ADL activities

## Score 0 = stable condition; Score 5 = highly unstable and in risk of death, hospitalization, pain, caregiver stress and poor self-rated health.

### Score 6 = much initiative and participates in social activities; Score 0 = severe withdrawal from social engagement

**Table 5:** Predictors of mortality (controlled for age and gender*) **

		95% Confidence Interval for Exp(B)		
	**Hazard ratio - Exp(B)**	**Lower**	**Upper**	**p-value**

ADL Long Scale^#^				< 0.001
1 = 0-3	1			
2 = 4-9	1.17	0.95	1.43	0.134
3 = 10-17	1.33	1.08	1.63	0.007
4 = 18-28	1.80	1.45	2.23	< 0.001
The Changes in Health Scale^##^				< 0.001
0	1			
1	1.18	0.98	1.41	0.079
2	1.61	1.35	1.93	< 0.001
3	2.16	1.70	2.75	< 0.001
4	3.95	3.08	5.07	< 0.001
5	16.18	11.41	22.95	< 0.001
Index of Social Engagement^###^				0.007
6	1			
5	1.36	0.94	1.97	0.102
4	1.19	0.86	1.65	0.303
3	1.32	0.96	1.81	0.092
2	1.49	1.09	2.04	0.013
1	1.62	1.19	2.22	0.002
0	1.63	1.22	2.19	0.001
Admitted from				0.011
Private home, with and without home help	1			
Board and care/assisted living/group home	1.11	0.86	1.45	0.417
Nursing home/nursing ward	1.09	0.88	1.37	0.408
Acute care hospital/ rehabilitation hospital	1.27	1.10	1.47	0.001

*Cox regression was performed controlling for age in four age groups (50-79, 80-84, 85-89, 90-104) and gender

**Variables entered into the Cox regression were: ADL Long Scale, CHESS, ISE, admitted from, pain scale, CPS and DRS.

# Score 0 = independent or only needs supervision; Score 28 = severe impairment in all four ADL activities

## Score 0 = stable condition; Score 5 = highly unstable and in risk of death, hospitalization, pain, caregiver stress and poor self-rated health.

### Score 6 = much initiative and participates in social activities; Score 0 = severe withdrawal from social engagement

## Discussion

This study showed the median survival time of nursing home residents in Iceland to be 31 months (2.6 years) with a stable death rate over the period of the study. Almost a third of the residents had died within a year from admission; a majority had died within 3 years, and less than half of the residents lived longer than 3 years. Those dying within the first year had less stable health, worse ADL performance, more pain, more depression and were less involved in social engagement. Significant predictors of mortality were age, gender, where admitted from, ADL functioning, health stability and social engagement.

The reported survival time in this study is similar to two recent studies with a 5 year follow-up time where the median survival of nursing homes was 2.3 years (N. Irel.; n = 2.112) [32] (US; n = 468) [33]. Other studies have reported higher [3] or lower [1] mean survival times. However, any cross-country comparison of survival times must take into account the availability of home care services and the criteria for nursing home placement in the respective countries; admission criteria for nursing home placement especially may complicate comparison.

Combined health stability and ADL performance seem to be valid predictors of mortality and should thus be considered when selecting a preferable type of service for older persons. These findings resemble those in other studies with regard to health stability [2,34] as well as ADL capacity [1,35]. In this study health stability, in particular a score of 2 or higher, was a strong predictor of mortality. Residents with this score were 1.61 times likelier to die during the investigation period, and those with a score of 5 were 16.12 times likelier to die. Also, a score of 10 or higher in ADL performance significantly predicted a higher risk of mortality than for those with a lower score. For instance those with a score 10-17 were 1.33 times likelier to die during the investigation period, and those with a score of 18-28 were 1.80 times likelier to die. Thus assessment of ADL and health stability seems to be helpful in selecting the most appropriate type of service. It may well be that older persons having a health stability score lower than 2 and an ADL score below 10 are better off in home care than nursing home placement. However, using only ADL capacity and health stability as a reference may be too narrow an approach. There may be other reasons for deciding on nursing home placement apart from those with increasing risk of mortality, such as difficult social circumstances or the person's mental health. Still it turned out that unstable health and low ADL capacity should be considered as important indicators of death and, in turn, more nursing care needs, such as services available at a nursing home.

It was noteworthy that low social engagement seems to be an important variable to take into account when predicting mortality. As a concept it may be viewed as the opposite of unstable health and low ADL capacity, as such debilitation would hinder a person from seeking or developing effective social engagement. The level of ADL capacity, however, does have some effect on the relationship of social engagement and survival time. In this study those with the least social engagement had an increased risk of death compared with the reference group who were deemed to have high initiative and participated in social activities. Those with a score of 2 were 1.51 times likelier to die than the reference group, and those with a score of 0, i.e. demonstrating severe withdrawal from social engagement, were 1.65 times likelier to die. Other studies have reported decreased social engagement to be a predictor of mortality for residents already living in nursing homes [3,36], rather than at admission. A study of one-year mortality of residents (US; n = 30.070) showed that greater levels of social engagement (scores 0-6 on the same scale as the present study) were associated with longer survival (p = 0.0001), and a one-point decrease in the index of social engagement meant that residents were 1.16 times as likely to die during the follow-up period [36]. The present study, however, revealed that only a score of 2 and lower in social engagement significantly predicted mortality, and the risk decreased with higher levels of engagement (Table 4). Causality cannot be established in the present study although it has been stated that social engagement influences residents well-being, and that social isolation may increase mortality and morbidity [37]. The nature of the relationship between social engagement and survival is complex. Social engagement may be hindered by disease and disabilities or other factors. Furthermore, environmental factors, activity and action by the individual may influence a person's health status [38]. Thus, it may well be that stimulating social engagement and individual activity may increase survival time.

The high percentage of residents dying in the first to third years of living in a nursing home suggests that the concept of palliative care may be a useful model for care in a nursing home. Research has furthermore indicated that increasing numbers of residents are dying in nursing homes instead of hospitals [4]. The findings of the present study suggest that one third of those admitted were already in a palliative stage at admission. Thus, the focus of nursing care in nursing homes needs to be on palliative care as much as restorative care. However, knowledge of palliative care and symptom management adapted to older people [4] as well as to those suffering from dementia is lacking in nursing homes [39].

The death rate was stable between cohorts, and in the first year after admission, it was 28.8% despite the fact that resources for nursing home care have decreased over the years. Findings from other studies differ and have reported both lower (17.5%) [3] and higher rates(34%) [2]. In a Swedish study on two cohorts (2001 and 2002) of old people (N = 626; 65-98 years) receiving public long-term care, the two-year mortality rate was 30% and 31%, respectively [35]. This was considerably lower than in the present study (43.4%). It should be noted that the Swedish subjects were receiving care at home as well as in nursing homes. However, where people were living was not an independent predictor of mortality [35]. Almost a third of the residents in the present study may have needed palliative care within a year of admission. These residents had less stable health, more ADL dependency, pain and depression and were less engaged socially - needs well within the concept of palliative care. Thus dying is a central issue in nursing care in nursing homes.

Although a majority died within a year in this study, 46.9% of the residents (n = 1035) lived longer than 3 years. They may have been detected prior to nursing home placement by systematic assessment of ADL capacity and health stability. Some of them may have benefitted from receiving a type of service other than nursing home placement. For instance, home care and rehabilitation might have delayed entry into nursing homes. Such an approach would have been more in line with the official policy of enabling older people to stay at home as long as possible. Enabling old people in relatively stable health and needing low levels of ADL assistance to stay at home longer would probably decrease the demand for nursing home placement.

The strength of this study is the inclusion of 11 cohorts and data based on residents' admission status. Registered nurses trained for the purpose performed the assessments, and only a valid instrument was used [22,40]. Nevertheless, this study has some limitations, such as variation in the sample of 13% to 84% of the total residents admitted each year to nursing homes [14]. The low percentage of the sample in the early years stems from the fact that these were the first years for mandatory assessment in all nursing homes in Iceland. It took some years for the assessment to be fully implemented, and in the early years residents were often not assessed until they had spent considerable time in the nursing home. Another limitation of concern is that the residents in the sample may have suffered some changes to their health after admittance and before being assessed. The delay in assessment is probably mostly related to workload and the absence of staff due to sickness or leaves rather than characteristics of the residents. The error should therefore be random rather than systematic. However, this can not be substantiated. Researchers have reported, however, a decline among nursing home residents over a six-month period [41] and a lower mortality risk of recently admitted residents compared to others [32]. The researchers' position, however, was that data from assessments within 90 days would sufficiently reflect the admission status of the residents.

Difference in mortality rates between nursing homes cannot be ruled out. It would have been preferable to investigate this, of course, but information on placement within individual nursing homes was not available. The reported significance of predictors of mortality may therefore vary in relation to nursing homes and this needs to be considered a limitation. Nursing homes in Iceland have however the same admission criteria and any difference in mortality rates are unlikely to have had a powerful effect.

## Conclusions

Health stability and ADL performance stand out as important predictors of mortality and would be appropriate to use not only at admission but also as a basis for deciding the appropriate service alternatives for older people in need of long-term care and service. A considerable number died within the first year, while others lived longer than 3 years in nursing homes. The latter group may have benefitted more from receiving home care and rehabilitation and thus might have deferred nursing home placement. The relatively short time a majority of residents lives in a nursing home implies that the concept of palliative care is useful as a model for nursing home care, in combination with restorative care. Knowledge of the course of development over the years in death rate and predictors of mortality seems important for health officials, managers and the nurses whose responsibility it is to plan and provide nursing care in nursing homes. Health assessment at admission and its implications in relation to predictors of mortality are valuable when planning individual care as well as nursing home services and staff knowledge.

## Competing interests

The authors declare that they have no competing interests.

## Authors' contributions

IH participated in the conception and design of the study, analysis and interpretation of data and was the main writer of the article. IRH participated in the conception and design of the study, writing of the article and critical revisions, analysis and interpretation of data and provided the principal oversight of the research. AKE and PN participated in the conception and design of the study, analysis and interpretation of data, oversight of the research, writing of the article and critical revisions. All authors read and approved the final manuscript.

## Pre-publication history

The pre-publication history for this paper can be accessed here:

http://www.biomedcentral.com/1472-6963/11/86/prepub
